# The international role of education in sustainable lifestyles and economic development

**DOI:** 10.1038/s41598-023-35173-w

**Published:** 2023-05-30

**Authors:** Xiangdan Piao, Shunsuke Managi

**Affiliations:** 1 Faculty of Humanities and Social Science, Iwate University, 3-18-34 Ueda, Morioka, Iwate 020-8550 Japan; 2 Urban Institute & Department of Civil Engineering, Kyushu University, 744 Motooka Nishi-Ku, Fukuoka, 819-0395 Japan

**Keywords:** Environmental economics, Sustainability

## Abstract

Improved economic growth and environmental protection are necessary to achieve the United Nations’ Sustainable Development Goals. This study examines the relationship between people’s education levels and sustainable lifestyles in protecting the environment and economic growth, as expressed by the increase in household equivalent income. We conducted an original cross-sectional survey, which yielded 100,956 valid observations in 37 countries. The factors included educational level, sustainable lifestyle with natural resource consumption, and household equivalent income for economic development. We used logit and ordered logit model and applied an ordinary linear regression model after confirming the association between education and income. Our analyses found that higher educational levels were associated with an increase in specific environmentally friendly behaviors and sustainable energy consumption. Individuals in the higher educational level group tended to consume recycled goods, purchase energy-saving household products, conserve electricity, and separate their waste. Additionally, higher levels of education were positively associated with equivalent household income in all 37 countries, indicating better economic development. Thus, our study underscores the importance of improving education at the broad population level to promote economic development and establish cooperative human behaviors necessary to sustain the environment.

## Introduction

In 2015, the United Nations proposed a list of 17 Sustainable Development Goals (SDGs), aimed at protecting the planet, with an implementation target set for the year 2030^1^. In the context of this study, notable goals are the elimination of poverty (goal 1), the establishment of good health and well-being (goal 3), reduced inequality (goal 10), responsible consumption and production (goal 12), and combating climate change (goal 13), all of which are crucial for human well-being. Human activity contributes significantly to the current environmental issues and causes the natural environment to undergo substantial changes, such as climate change. Thus, reshaping human activity toward economic and ecological sustainability is attracting increasing scholarly attention.

The higher educational institute, the university, plays a crucial role in the cities toward sustainable development due to the deep connection between the higher education institute and the local region. The universities are believed to be closely related to the cities’ hands-on city activities, entrepreneurship, sources of employment, innovation, and cooperation, etc.; the Leal^2^ analysis the university cooperation has favorable aspects on connecting sustainable cities, the remarkable elements that university contributes to the local cities are associated with the city government, local companies, NGOs; and activate the joint events or projects; moreover, the role of the universities strengthens the cooperation with the cities toward sustainability. Education is a central factor in human capital theory^3,4^. Studies have shown that education plays a critical role in economic growth and favorable labor market outcomes^3–5^. The United Nations proposed the 17 sustainable development goals that D’Adamo^5^ tries to introduce an alternative methodology to aggregate the indicators to present the economic, social, and environmental in the sustainable process. It is believed that human capital and the natural environment are complementary from the weak sustainability perspective. Based on the statistics derived from the national statistical offices of Italy, the results show that South Italy has suffered from the perspective of social-economic point, whereas the environmental performance is remarkable. Furthermore, sustainable education holds the young generation more aware of sustainable attitudes to the natural environment conservation and more confidence in structuring the future society^6^. To achieve the sustainable educational institutes, the universities should encourage the young generation involve in the project relates to the world.

By contrast, substantial environmental issues are closely associated with human activity, and pro-environmental behavior is encouraged in pursuit of ecological sustainability^7–9^. Attempts to prompt people toward environmental conservation are investigated within the framework of pro-environmental behavior. Pro-environmental behaviors comprise actions, such as saving energy, transportation selection, purchasing energy-saving goods, reducing waste, and recycling^10–12^. Similarly, “lifestyle” is defined as a cluster of habits and patterns within social networks that minimizes natural resources and reduces waste. Accordingly, a growing body of literature examines the factors influencing the likelihood of people engaging in pro-environmental behaviors^10–14^.

Focusing on both developed and developing countries, the proceeding literature investigates the relationship between human capital, economic growth, and natural environment conservation and types of energy consumption from a national perspective toward sustainable development^15–22^, for example, 17 OECD countries by Mujtaba et al.^19^; 16 European countries by Bekun et al.^16^; the Next Eleven (N-11) countries (Bangladesh, Egypt, Indonesia, Iran, Mexico, Nigeria, Pakistan, the Philippines, South Korea, Turkey, and Vietnam) by Rahim et al.^22^; 73 developing countries by Jahanger et al.^18^; and Brazil, China, India, and Russia by Nathaniel et al.^23^. However, the relationship between human capital and natural environmental conservation as illustrated in different countries is yet to be clarified.

Danish et al.’s^24^ analyses of the impact of human capital improvement on the natural environment in Pakistan shows a negative association between human capital improvement and natural environment conservation in the short-term. Moreover, they suggest that investment in population education improves awareness of protecting the natural environment. Jahanger et al.^18^ demonstrate the effect of technological innovation, human capital, economic growth on environment conservation, focusing on data derived from 73 developing countries located in Asia, Africa, and Latin American and Caribbean from 1990 to 2016. The empirical evidence shows that technological innovations has a moderate effect on environment conservation associated with natural resource consumption. The relationship between human capital and the natural environment in terms of renewable energy consumption is investigated based on the data derived from China, by Pata and Caglar^25^. They highlight the long-term favorable effect of human capital on natural environmental degradation. In South Africa, Iorember et al.^26^ confirm the positive influence of human capital and natural environment quality both in the short-term and long-term.

In India, Ahmed and Wang^27^ illustrate the impact of human capital on the ecological footprint from 1997 to 2014, and they highlight the significant negative association between human capital and ecological footprint. They recommend policies to develop human capital in developing nations. In sum, education investment associated with technology growth improves public awareness of natural environment conservation. By contrast, Kassouri and Altintas^28^ show that in MENA countries, the negative trade-off relationship between ecological footprint and human well-being is captured by human capital development from 1990 to 2016.

On the one hand, efforts to undo some of those damages have led to the establishment of a sustainable lifestyle, which is defined as “a cluster of habits and patterns of behavior embedded in a society and facilitated by institutions, norms, and infrastructures that frame individual choice, in order to minimize the use of natural resources and generation of waste, while supporting fairness and prosperity for all” according to Akenji and Chen^13^. Many studies have focused on the effects of such a lifestyle, showing several positive outcomes^29–36^. Notably, this lifestyle requires higher levels of education^37^. On the other hand, standardized solutions to improve human activities aimed at environmental preservation and economic development are lacking. Regarding the determinant factors that enhance people’s pro-environmental behavior, knowledge of environmental issues, educational attainment, family structure, childhood experiences, age, psychological well-being, and life satisfaction are significantly associated with pro-environmental behavior. Factors, such as their household income, household size, cultural values, green advertisements, internal psychological scales for altruism, environmental attitudes, knowledge of environmental issues, and beliefs have also been found to be significant^38–49^. Moreover, a detailed review of determinants of pro-environmental behavior include education, environment conservation attitudes, culture, and values.

However, a cross-country relationship or the national perspective effect of the human capital to the economic growth is shown based on the analysis of national data. It indicates a cross-country correlation of natural environment conservation and population education improvement. It is known that a cross-country relationship is difficult to represent the complex within country situations; therefore, the micro data derived at the individual level may provide a different viewpoint of within-country relationships between human capital, environment conservation, and economic growth. Owing to data limitations, the individual level analysis of the comprehensive relationship between education, income, and environment conservation is scarce. This study tries to answer the research question that whether or not individuals’ education attainment and income are positively associated both in high- and low-income countries. And whether or not individuals’ education attainment has a favorable effect on household sustainable lifestyle both in high- and low-income countries.

This study extends current knowledge based on the following aspects. This study focuses on the relationship between education, sustainable lifestyle, and household income, using large-scale (around 100,000) individual-level data derived from 37 nations. First, results demonstrate the need to alter the rate at which humans consume natural resources and investigate the educational levels and sustainable lifestyle elements in 37 nations, thus covering approximately 73% of the world’s population. Second, previous studies using microdata have mainly focused on either economic growth or human activities for environmental conservation. However, few studies examine both economic and environmental sustainability using individual-level data. People’s pro-environmental behavior is closely related to environmental issues, whereas economic growth is related to employment and family income. From the individual’s perspective, therefore, we provide important insights for policies aimed at changing attitudes for a more efficient consumption of natural resources.

This study aimed to investigate the relationship between individuals’ educational levels and sustainable lifestyles to confirm the role of education on people’s pro-environmental behavior toward reducing natural resource consumption. In addition, the assessment was conducted in both high- and low-income countries to show heterogenous associations. Furthermore, the role of education on household income and sustainable lifestyles was examined for each of the 37 countries to illustrate economic growth and resource consumption and to show the universal and estimated relationship between them. The empirical analysis was based on an internet survey and face-to-face surveys of the population in regionally and culturally representative countries. People’s sustainable lifestyles include recycling goods, electricity conservation, energy-saving products, and waste separation/avoidance. These types of lifestyles are closely related to environmental issues, and economic growth is related to employment and family income.

## Methodology and data

### Methodology

Regarding the achievement of sustainable economic development and environmental preservation, we investigated the relationship between sustainable lifestyle behaviors and educational attainment levels (Eq. 1). Referring to the econometric theory^50,51^, when the dependent variable is a binary variable, the logit model and the probit model is believed to be appropriate. Consistent with the previous applied regression model section in Chapman et al.^52^, the logit model is adopted. Equation (1) was constructed as follows:

1 $$ln(\frac{{P}_{iC}}{1-{P}_{iC}})={\theta }_{0}+{K}_{iC}^{\mathrm{^{\prime}}}\theta +{X}_{iC}^{\mathrm{^{\prime}}}\beta +{D}_{C}^{^{\prime}}\delta$$

where ${P}_{iC}=prob({S}_{iC}=1)$ is individual $i$’s probability of being involved in sustainable lifestyle behaviors. A sustainable lifestyle includes recycled goods usage, electricity conservation, the purchase of energy-saving products, and sorting/reducing waste in the country $C$. We separately conducted regressions for each sustainable lifestyle behavior. The $K$ denotes a set of educational attainments (senior high school, vocational school, university, and graduate school). The $X$ represents socioeconomic and demographic factors, including age, occupational status (full-time employee, business owner, professional worker, self-employed, homemaker, part-time employee, government employee, student, unemployed, and others), housing status (own, rent, and others), female dummy (yes = 1, no = 0), presence of children dummies (no child, one child, two children, and three or more children). The set of dummy variables $D$ was used to control for country-specific heterogeneity. ${\theta }_{0}$, $\theta$, $\beta$ and $\delta$ are estimated parameters. The error term was denoted as $\varepsilon$.

Human capital is known to play a crucial role in economic development^53,54^, and educational attainment is a key factor for human capital. As the natural environment continues to change, it becomes increasingly important to focus on both economic development and environmental preservation. In this context, sustainable lifestyle behaviors are highly important for environmental preservation, especially at the individual level^9^. As such, this study investigated the relationship between educational attainment and sustainable lifestyle behaviors, aimed at both economic development and environmental preservation. It was found that, if the coefficients for educational attainment are positive, higher educational levels are positively associated with increased sustainable lifestyle behaviors, such as the purchase of energy-saving products.

As the impact of education on sustainable lifestyle may vary country-wise, we accounted for country-specific heterogeneity in the relationship between education and a sustainable lifestyle by running separate regressions for each country. According to the econometric theory^50,51^, when the dependent variable is the scaled dependent variable, the ordered logit model or ordered probit model is appropriate. Thus, based on prior studies, our empirical analysis adopted the model^55,56^. This study used the ordered logit model in the main results and the ordered probit model to verify robustness^51,52,55,56^. For this purpose, we constructed Eq. (2) as follows:

2 $${H}_{i}=\rho +{\gamma L}_{i}+{X}_{i}^{^{\prime}}\eta +{\varepsilon }_{i}$$

in which sustainable lifestyle is denoted as $H$, with greater numbers indicating better lifestyles for environment sustainability. The year of education variable is denoted as $L$, which was derived from these educational attainment levels: never attended school = 0, dropped out of primary school = 3, completed primary school = 6, completed junior high school = 9, completed high school = 12, completed vocational school = 14, completed junior college = 15, completed university/college = 16, completed graduate school [master’s degree] = 18, and completed graduate school [doctoral degree] = 23). The $X$ represents socioeconomic and demographic variables, including age, occupational status (full-time employee, business owner, professional worker, self-employed, homemaker, part-time employee, government employee, student, unemployed, and others), housing status (own, rent, and others), female dummy (yes = 1, no = 0), presence of children dummies (no child, one child, two children, and three or more children). The values of $\rho$, $\gamma$ and $\eta$ are estimated parameters.

Finally, we constructed Eq. (3) to investigate the impacts of education on economic development, using the ordinary least square model:

3 $${I}_{i}=a+{bL}_{i}+{X}_{i}^{^{\prime}}c+{\varepsilon }_{i}$$

in which the continuous dependent variable ${I}_{i}$ is the individual’s logarithm of household equivalent income. Variable ${L}_{i}$ denotes individual $i$’s education level, with greater numbers indicating more years, (i.e., never attended school = 0, dropped out of primary school = 3, completed primary school = 6, completed junior high school = 9, completed high school = 12, completed vocational school = 14, completed junior college = 15, completed university/college = 16, completed graduate school [master’s degree] = 18, and completed graduate school [doctoral degree] = 23). The $X$ again represents socioeconomic and demographic variables, including age, occupational status (full-time employee, business owner, professional worker, self-employed, homemaker, part-time employee, government employee, student, unemployed, and others), housing status (own, rent, and others), female dummy (yes = 1, no = 0), presence of children dummies (no child, one child, two children, and three or more children). Parameters a, $b$, and $c$ were estimated parameters. When educational levels improve the income levels, parameter $b$ is expected to have a positive value and statistical significance.

The relationship between the sustainable lifestyle behaviors and educational attainment levels using a logit model and ordered logit model, whereas the association between education and income is explored using ordinary least square models by controlling the individuals’ social and demographic background for the 37 nations using the original survey. The strength of the analysis using original survey provides a clear picture of the international viewpoint of this issue with the consistent analysis method.

### Data

To measure the aggregate wealth of society toward sustainability, we conducted an original cross-sectional survey, including 37 nations across six continents, through a third-party survey company in Japan (Nikkei Research Company; interviews were conducted both via the internet and face-to-face). The targeted respondents were selected randomly to match the population’s age and gender distribution. Surveys were individually conducted in the investigated countries from 2015 to 2017, yielding 100,956 valid observations. Respondents provided detailed information on their socioeconomic backgrounds, subjective well-being, environmentally sustainable activities, and socioeconomic inequality. The survey was administered in the following countries, arranged by continent, via internet surveys:

1. China, India, Indonesia, Japan, Malaysia, Singapore, Thailand, the Philippines, and Vietnam.
2. Czechia, France, Germany, Greece, Hungary, Italy, the Netherlands, Poland, Romania, Russia, Spain, Sweden, Turkey, and the United Kingdom.
3. Canada, Mexico, and the United States
4. Brazil, Chile, Colombia, and Venezuela.
5. Australia.
6. South Africa.

Face-to-face surveys were conducted in five countries, namely Egypt, Kazakhstan, Mongolia, Myanmar, and Sri Lanka, where internet-based surveys were not feasible.

The above 37 countries contain approximately 73% of the world’s population. Detailed observations for each country and additional information on the data collection period are available in the appendices.

### Variable selection

#### Sustainable lifestyle factors and educational attainment

We selected the following comprehensive activities that are closely related to populational energy consumption and waste recycling. This resulted in a multidimensional range of relevant activities. The questionnaires prompted respondents with the following: “Please select all actions that you have taken these days. Also, please select all activities that you have participated in these days”:

1. Purchasing recycled goods.
2. Energy-saving actions (saving electricity, fuel, etc.).
3. Purchasing energy-saving household products.
4. Recycling or sorting waste/reduction of waste.

Respondents rated all items using a binary code: yes = 1, no = 0. Finally, we constructed a comprehensive sustainable lifestyle variable based on an unweighted summation of the above items. This sustainable lifestyle variable thus ranged from 0 to 4 and was expected to capture overall individual activity levels aimed at environmental sustainability, with higher values indicating increased environmental friendliness.

Regarding educational attainment, respondents reported their backgrounds as follows: junior high school or lower, senior high school, vocational school, university, and graduate school. As education is a key factor in economic development^56–62^, education dummies were used to investigate the relationship between educational attainment and individual-level activities aimed at environmental preservation. Years of education were derived from these educational attainment levels: never attended school = 0, dropped out of primary school = 3, completed primary school = 6, completed junior high school = 9, completed high school = 12, completed vocational school = 14, completed junior college = 15, completed university/college = 16, completed graduate school [master’s degree] = 18, and completed graduate school [doctoral degree] = 23.

#### Other explanatory variables

Other explanatory variables included the following: (1) household income, (2) age, (3) occupational status (full-time employee, business owner, professional worker, self-employed, homemaker, part-time employee, government employee, student, unemployed, and others), (4) housing status (own, rent, and others), (5) female dummy (yes = 1, no = 0), (6) presence of children dummies (no child, one child, two children, and three or more children), and (7) dummies for each country.

In the above context, household income was divided by the square root of the number of family members and then transformed into equivalent household income. Amounts were converted into US dollars using the exchange rate as of January 7, 2021. The equivalent scale is the square root of the number of families.

### Ethics approval

For the original cross-sectional internet survey conducted by a third-party company (Nikkei Research Company) between 2015 and 2017, the study design was approved by the appropriate legal and ethics review board of Kyushu University. The data were collected with informed consent from participants, according to legal and ethical guidelines. All the methods proceeded in accordance with ethical guidelines and were approved by the ethical committee of Kyushu University.

## Results

**Figure 1:**
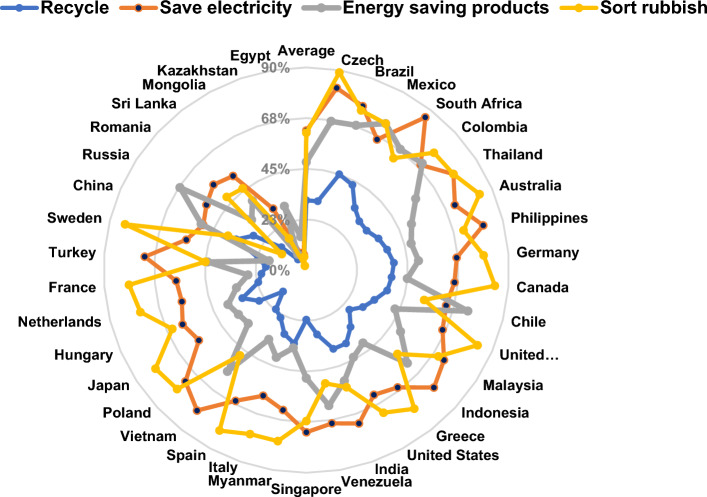
Multinational rankings for sustainable lifestyles in 37 nations. Data were obtained through the original survey conducted in this study. Rankings are based on the unweighted summation of recycled goods, electricity conservation, the purchase of energy-saving products, and sorting/reducing rubbish. *Source* Authors’ calculations.

From the national perspective, the descriptive statistics and sustainability rankings for individual-level lifestyles, including recycling, electricity conservation, use of energy-saving products, and waste separation/avoidance (overall averages of 31%, 62%, 48%, and 61%, respectively), are shown for all 37 countries surveyed (Fig. 1). The rankings are based on the unweighted sum of recycling, saving electricity, buying energy-saving products, and waste separation/reduction. The ranking ranges from Czechia (1st place) to Egypt (37th place). These results highlight the substantial potential for improvement in multiple areas. Examining specific countries showed that China has the world’s largest population but ranks 31 among 37 countries. Therefore, improvements targeted at sustainable consumption, waste reduction, and/or energy conservation may constitute a critically important contribution to global environmental preservation. However, results showed that individuals in developing countries were more likely to save electricity and manage waste, which may be reflective of general income constraints.

Overall, the results of this study indicate the positive relationship between educational attainment and a sustainable lifestyle, and the positive association between educational attainment and income is confirmed. As shown in Table 1, notable results were seen. First, university-level or higher education individuals were more likely to engage in sustainable activities. In column (1), the coefficients for senior high school, vocational school, university, and graduate school were 0.028, 0.042, 0.061, and 0.076, respectively; statistical significance was established at 1%, with the reference group being junior school or lower. These results suggest that people with educational attainment have a higher proclivity to consume recycled goods than those with less or no education, even when other control variables remain constant (a magnitude ranging from 2.8% to 7.6% according to educational attainment). As shown in column 4, we found similar results for sorting/reducing waste; in particular, individuals with master’s or doctoral degrees were 13.4% more likely to sort/reduce waste compared with those in the lower education group. This may be because those with more extensive education have increased knowledge about the importance of environmental preservation, which influences them to engage in environmentally friendly behaviors.

**Table 1:** Impact of individual socioeconomic background on sustainable lifestyle in 37 nations.

Variables	Recycled goods	Save electricity	Energy-saving products	Sort/reduce waste
	(1)	(2)	(3)	(4)
	Coeff	Coeff	Coeff	Coeff
Educational attainment				
(ref. junior school or lower)				
Senior high school	0.028***	0.101***	0.080***	0.078***
	(0.006)	(0.007)	(0.007)	(0.007)
Vocational school	0.042***	0.127***	0.108***	0.104***
	(0.007)	(0.008)	(0.008)	(0.008)
University	0.061***	0.148***	0.121***	0.118***
	(0.006)	(0.006)	(0.007)	(0.007)
Graduate school	0.076***	0.137***	0.098***	0.134***
	(0.007)	(0.008)	(0.008)	(0.008)
ln (household income)	0.011***	0.008***	0.014***	0.023***
	(0.002)	(0.002)	(0.002)	(0.002)
Occupational status				
(ref. unemployed)				
Full-time employee	0.042***	− 0.015**	0.017**	− 0.004
	(0.006)	(0.007)	(0.007)	(0.007)
Part-time employee	0.054***	0.004	0.038***	0.042***
	(0.008)	(0.008)	(0.009)	(0.009)
Company owner	0.021*	− 0.041***	− 0.020	− 0.050***
	(0.012)	(0.013)	(0.014)	(0.014)
Government employee	0.019*	0.015	0.028**	− 0.037***
	(0.011)	(0.011)	(0.012)	(0.012)
Professional	0.058***	0.038***	0.048***	0.005
	(0.010)	(0.011)	(0.011)	(0.012)
Self-employed	0.060***	0.022**	0.055***	0.015
	(0.008)	(0.009)	(0.009)	(0.009)
Student	0.050***	0.049***	0.033***	0.061***
	(0.009)	(0.010)	(0.011)	(0.011)
Homemaker	0.022***	0.027***	0.066***	0.018**
	(0.008)	(0.009)	(0.009)	(0.009)
Other	0.005	0.007	0.027***	0.026***
	(0.008)	(0.009)	(0.009)	(0.009)
Age	− 0.001***	0.004***	0.003***	0.003***
	(0.000)	(0.000)	(0.000)	(0.000)
Housing status (ref. renter)				
Owner	− 0.001	0.017***	0.037***	0.030***
	(0.004)	(0.004)	(0.004)	(0.004)
Other	− 0.058***	− 0.067***	− 0.033***	0.002
	(0.010)	(0.010)	(0.011)	(0.011)
Number of children				
(ref. no child)				
One	0.054***	0.038***	0.061***	0.024***
	(0.004)	(0.005)	(0.005)	(0.005)
Two	0.050***	0.030***	0.066***	0.032***
	(0.005)	(0.005)	(0.005)	(0.005)
Three or more	0.071***	0.050***	0.079***	0.031***
	(0.006)	(0.006)	(0.006)	(0.007)
Female dummy	0.036***	0.030***	0.008**	0.035***
	(0.003)	(0.003)	(0.004)	(0.004)
Country dummies	YES	YES	YES	YES
Observations	92,128	92,128	92,128	92,128

*Source* Authors’ calculations.

Standard errors in parentheses. *** *p* < 0.01, ** *p* < 0.05, * *p* < 0.1. (1) definition of dependent variables: (a) whether individuals purchase recycled goods (yes = 1, no = 1), (b) individuals use energy-saving household products (yes = 1, no = 0), (c) purchase energy-saving household products (yes = 1, no = 0), and (d) sort or reduce waste (yes = 1, no = 0). Model: logit model.

Regarding electricity conservation and the purchase of energy-saving products, respondents with a university education had the highest level of awareness regarding the benefits of environmentally friendly behaviors pertaining to energy consumption. As shown in column (2), the coefficients for senior high school, vocational school, university, and graduate school were 0.101, 0.127, 0.148, and 0.137, respectively, which indicates that individuals with higher education levels engage in electricity-saving activities at 10.1% to 14.8% higher rates. We found similar results for purchasing energy-saving products, where highly educated individuals were 12.1% more likely to do so. In summary, individuals with university-level education reported more environmentally friendly lifestyles.

Second, higher household income was associated with energy conservation and sorting/reducing waste. As shown in columns (1) through (4) of Table 1, the coefficients for household income were positive and statistically significant. In other words, individuals with higher household incomes were more likely to engage in sustainable lifestyle behaviors, including sorting/reducing waste (highest impact), purchasing energy-saving products, consuming recycled goods, and conserving electricity (lowest impact).

Other socioeconomic and demographic factors also influence sustainable lifestyle behaviors. First, women were more likely to engage in these behaviors compared with men (statistically significant at 1%). On average, women were 3.6% more likely to consume recycled goods, 3% more likely to conserve electricity, 0.8% more likely to purchase energy-saving products, and 3.5% more likely to sort/reduce waste. Second, homeowners were more likely to engage in environmental preservation compared with renters and others. Third, occupation status impacted sustainable lifestyle behaviors, as full-time employees and business owners were less likely to conserve electricity compared with unemployed individuals. In addition, business owners and government employees were not likely to sort/reduce waste. Fourth, households with children tended to conserve energy and use environmentally friendly goods.

Based on our logit model (see Eq. (1)), Table 2 shows the relationship between educational attainment and sustainable lifestyle behaviors in higher- and lower-income countries. The dependent variables were use of recycled goods, electricity conservation, purchase of energy-saving products, and sorting/reducing waste. The major independent variables were educational levels, that is, dummy variables comprising senior high school, vocational school, university, and graduate school. The results for higher- and lower-income countries are shown in Panels A and B, respectively.

**Table 2:** Relationship between education and sustainable lifestyles for high- and low-income countries. Standard errors are shown in parenthesis.

A: High-income countries	(1)	(2)	(3)	(4)
Variables	Recycled goods	Save electricity	Energy- saving products	Sort/reduce waste
	Coeff	Coeff	Coeff	Coeff
Education attainment				
(ref. Junior school or lower)				
Senior high school	0.025***	0.095***	0.054***	0.076***
	(0.009)	(0.009)	(0.009)	(0.007)
Vocational school	0.040***	0.112***	0.080***	0.094***
	(0.010)	(0.010)	(0.011)	(0.008)
University	0.049***	0.120***	0.075***	0.097***
	(0.008)	(0.008)	(0.009)	(0.007)
Graduate school	0.068***	0.115***	0.042***	0.088***
	(0.010)	(0.010)	(0.011)	(0.008)
ln (household income)	0.010***	0.024***	0.025***	0.023***
	(0.003)	(0.003)	(0.003)	(0.003)
Country dummy	YES	YES	YES	YES
Observation	43,274	43,274	43,274	43,274

B: Low-income countries	(5)	(6)	(7)	(8)
Variables	Recycled goods	Save electricity	Energy- saving products	Sort/reduce waste
	Coeff	Coeff	Coeff	Coeff
Education attainment				
(ref. Junior school or lower)				
Senior high school	0.026**	0.094***	0.095***	0.030***
	(0.010)	(0.010)	(0.011)	(0.011)
Vocational school	0.045***	0.137***	0.132***	0.055***
	(0.012)	(0.012)	(0.012)	(0.013)
University	0.070***	0.165***	0.159***	0.084***
	(0.009)	(0.009)	(0.010)	(0.010)
Graduate school	0.090***	0.158***	0.161***	0.132***
	(0.011)	(0.012)	(0.012)	(0.013)
ln (household income)	0.013***	− 0.000	0.010***	0.022***
	(0.002)	(0.003)	(0.003)	(0.003)
Country dummy	YES	YES	YES	YES
Observation	48,854	48,854	48,854	48,854

*Source* Authors’ calculations.

*** *p* < 0.01, ** *p* < 0.05, * *p* < 0.1. Model: Logit model.

In both high- and low-income countries, educational attainment had favorable effects on household energy consumption and waste reduction. For example, as shown in column (3), the coefficient for “university” was 0.120, which suggests that individuals with a university-level education were 12% more likely to engage in energy-saving activities when compared with individuals with lower levels of education. Consistent results were found for all other educational levels and sustainable lifestyle activities. The coefficients for the education attainment dummies were positive in all the cases (senior high school through graduate school), ranging from 0.025 to 0.120 in higher-income countries and from 0.026 to 0.161 in lower-income countries. This also shows that the positive effects of educational attainment were more pronounced in lower-income countries, which may be related to the condition that improved population education especially facilitates economic development in lower-income countries.

**Figure 2:**
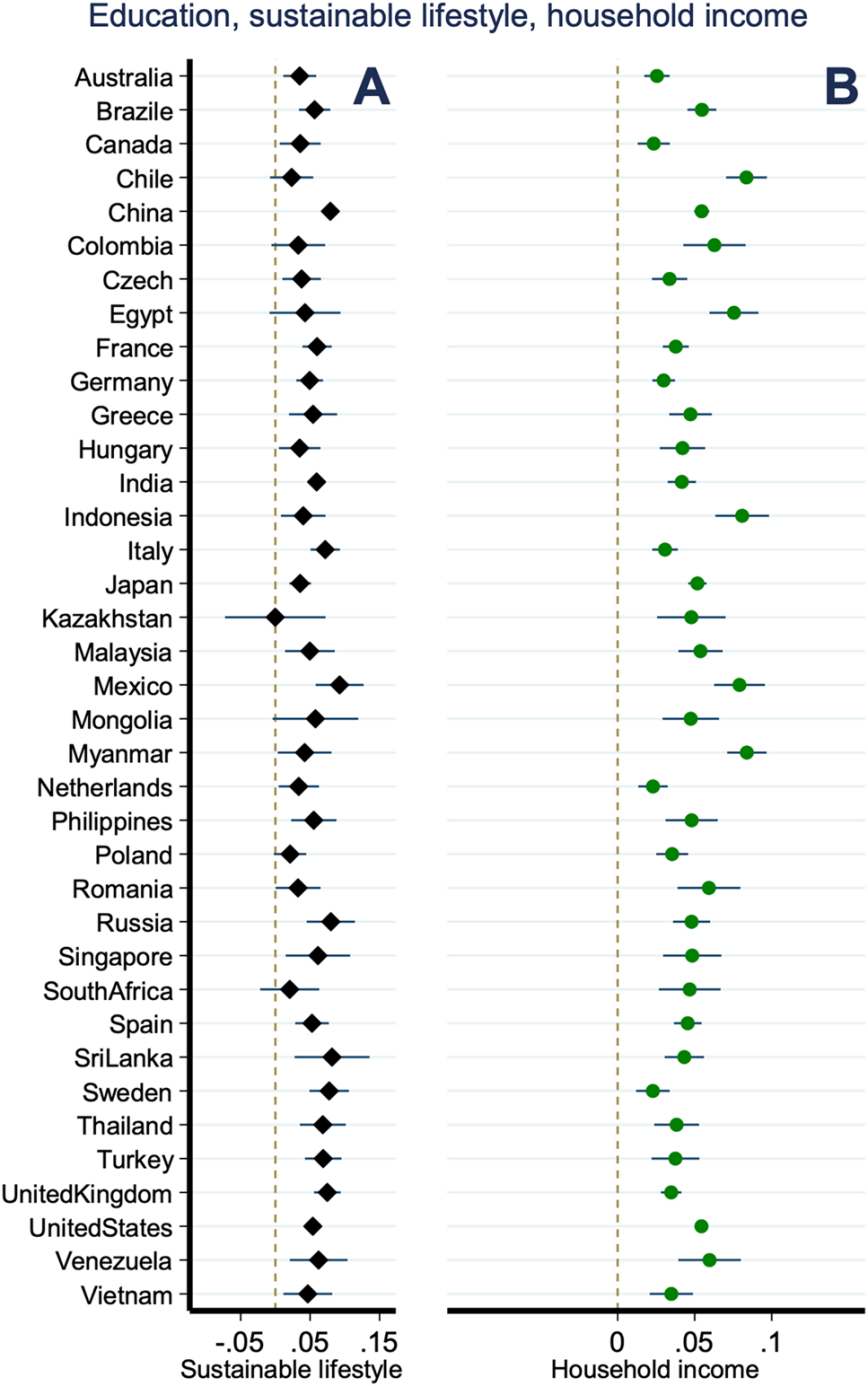
Impact of education on sustainable lifestyles and household income for each surveyed country. Lines denotes a 95% confidence interval. “Filled diamond” denotes the coefficient of schooling by regressing the sustainable lifestyle on schooling and other explanatory variables using an ordered logit model. “Filled circle” denotes the coefficient of schooling by regressing the logarithm of the household equivalent income on schooling and other covariates using an ordinary least square model. Dashed Lines denotes the zero. Source: Authors’ calculations.

Based on our ordered logit model (see Eq. (2)), Fig. 2 shows the influence of increased education on potential improvements in environmentally friendly activities in each surveyed country. The dependent variable was set as the transformed sustainable lifestyle index, which is the unweighted summation of sustainable activities, including the purchase of recycled goods, electricity conservation, the use of energy-saving household products, and sorting/reducing waste. Here, higher values indicate better sustainable lifestyle behaviors. We also analyzed the association between education and economic development for each country via an ordinary least squares model (see Eq. (3) in the Methods section), as shown in panel B of Fig. 2. The value of education was calculated as the years of education, whereas higher education was denoted as more years of schooling.

The results clearly show that higher education levels were associated with increased sustainability behaviors aimed at environmental preservation. In general, this suggests that education is a crucial factor not only for economic development but also for environmental protection. The coefficients for years of education ranged from 0 in Kazakhstan, to 0.82 in Sri Lanka. In 33 of the 37 countries surveyed, higher education levels were statistically and significantly associated with higher frequencies of environmentally friendly behaviors. The said 33 nations include Australia, Brazil, Canada, China, Colombia, Czechia, France, Germany, Greece, Hungary, India, Indonesia, Italy, Japan, Malaysia, Mexico, Mongolia, Myanmar, the Netherlands, the Philippines, Poland, Romania, Russia, Singapore, Spain, Sri Lanka, Sweden, Thailand, Turkey, the United Kingdom, the United States, Venezuela, and Vietnam. From the results of the analysis, the preferred education effect was estimated based on the original survey data; for example, China and India have the world’s two largest populations, which emphasizes that considerably large environmental improvements can be achieved through increased populational education (respective magnitudes of 0.079 and 0.059, statistically significant at 1%).

As mentioned, higher education is positively associated with household equivalent income. The coefficients for years of education were positive, ranging from 0.023 in Canada, the Netherlands, and Sweden, to 0.084 in Chile and Myanmar (statistically significant at 1%). The results also reveal that one year’s additional education produced income increases ranging from 2.3% to 8.4%. In summary, national efforts to increase populational education should help achieve better economic development while increasing the overall level of environmental protection. In this regard, our results have important national policy implications for countries working toward the United Nations’ SDGs and striving for better economic development.

## Discussion

How does education influence individuals’ sustainable lifestyles and economic growth from an international perspective? Many scholars and policymakers are now focused on achieving sustainable economic development, while also preserving the natural environment. Therefore, sustainable lifestyle behaviors are crucial, although often difficult to implement. This study clarifies the frequencies of specific behaviors by having conducted a survey spanning 37 nations across six continents, resulting in more than 100,000 international observations. Our subsequent analyses produced some important insights.

First, data from the higher educational institutions shows cities’ considerable efforts toward sustainable development owing to the cooperation between higher educational institutions and local governance bodies through employment and innovation^6,63^. Educational attainment played a crucial role in increasing sustainable lifestyle behaviors, specifically in the context of using recycled goods, purchasing energy-saving products, conserving electricity, and reducing/sorting waste. For example, individuals with higher educational levels (e.g., graduate degrees) were 14.8% more likely to engage in electricity conservation behaviors compared to those with lower education levels (e.g., junior high school level). This proved true even when other socioeconomic and demographic factors remained constant. Similar trends were observed for recycling, energy-saving products, and sorting/reducing waste, with magnitudes ranging from 7.6% to 13.4%. This indicates that higher educational levels are positively associated with favorable lifestyle behaviors aimed at environmental preservation. On average, respondents used recycled goods, conserved electricity, purchased energy-saving household products, and sorted/reduced waste at 31%, 62%, 48%, and 61%, respectively. The positive association between educational attainment and a sustainable lifestyle is consistent with previous studies^18,25–27^.

Achieving natural environment sustainability requires fostering a sustainable lifestyle and sustainable consumption production. Higher educational institutions are crucial in promoting better governance and joint city activities between public activities organized through collaboration between local governance bodies and higher educational institutions, while adopting innovation and cooperation^2,6,63^. Consistent with the previous studies, encourage the young generation participate real world associated project improves the social activation for the sustainability.

Second, the relationship between human capital and environment conservation behavior in various countries has been confirmed. For example, the short-tern positive association is confirmed in Pakistan^24^, a long-term positive impact of the human capital on the natural environment conservation is found in China^25^, and both long-term and short-term favorable impact are seen in South Africa. In this study, comparing the human capital increase in higher and lower-income countries revealed that education substantially affects sustainable lifestyle behaviors. More favorable effects were seen in lower-income countries: individuals with higher levels of education were 9% to 16.5% more likely to engage in sustainable energy conservation and waste sorting/reduction activities. These results are consistent with previous studies that focused on pro-environmental behavior^10,11^. In this context, improved populational education is expected to help sustain natural resource consumption, especially in lower-income countries.

Third, based on the individual-level microdata from each country, higher education levels were found to be associated with increased sustainability behaviors aimed at environmental preservation. This result suggests that education is a crucial factor for economic development and environmental preservation. We found a preferred education effect in 32 of 37 nations, including China and India, which have the world’s first and second largest populations, respectively; this shows that profound effects can be achieved by enhancing education at the populational level.

Furthermore, we found a positive relationship between educational attainment and increased income in all 37 nations studied. The results reveal that one additional year of education improved annual incomes by 2.3% to 8.4%. This finding is consistent with previous studies, such as Rahim et al.^22^, that disclosed that human capital and natural resources contribute to a positive impact on economic growth. By contrast, the positive relationship between economic growth increases the burden on natural resources; however, no causal relationship is confirmed. In South Africa, the unfavorable casual impact of the economic growth on the natural environment is seen in Iorember et al.^26^. In India, the negative u-shaped pattern between economic growth and the natural environment burden is confirmed^27^. Overall, as there are clear positive impacts on environmental preservation, large-scale efforts to improve populational education may enhance sustainability practices at the societal level.

As indicated by the results, the policy implications for all the investigated countries are as follows. First, widespread education should increase the frequency at which individuals engage in activities aimed at environmental preservation, which is critically important for responding to current changes in the natural environment. This corroborates evidence produced in previous studies on the need to reshape human behavior toward more sustainable resource usage^9^. Second, economic development is closely associated with daily household life. In this case, economic development and natural preservation are core, intertwined issues, both in the short-and long-term. Our results clearly show that education is a crucial factor for not only economic development but also environmental preservation; that is, higher education is associated with more environmentally friendly behaviors at the individual level. This effect was more pronounced in lower-income countries, including those with substantially large populations, such as China, India, and Indonesia. In summary, policymakers may take these factors into consideration by implementing policies aimed at increasing population education; this will contribute to both better economic development and environmental conservation, as education clearly increases the rate of sustainable behavior. To produce sustainable educational institutes, universities could offer more encouragement to the younger generation involved in project solving of real-world issues.

This study clarifies the relationship between educational level and sustainable lifestyle, as well as household income, from an international perspective. However, this study is subject to some limitations. First, this study used the original internet and face-to-face survey data collected by Nikkei Research. A focus on internet users in research is more likely to target those with better education or higher income, which might result in a sample selection bias in the regression analysis. We attempted to conduct face-to-face surveys in some nations to perform robustness checks, and we found the results to be consistent with the sample derived from the internet survey done by Nikkei Research. However, the main results might still exhibit a sample selection problem; therefore, future studies are encouraged to employ comprehensive datasets to investigate this issue. Second, we examined the relationship between education and economic growth as well as sustainable lifestyles in this study, considering cross-sectional international survey data from 37 nations. Future studies could further explore the causal effect of education on economic growth and sustainable lifestyles. Furthermore, although this study confirmed a positive relationship between education and sustainable lifestyles as well as household income, the results do not explain whether it is a casual effect; this could be examined in future studies. This study uses original internet and face-to-face data derived from 37 nations during 2015 to 2017. The original survey collected detailed individual level environmental conservation attitudes, pro-environmental behavior, and socio-economic and demographic background, allowing this study to investigate the relationship between education, economic growth, and environment conservation from individual perspectives. The results of this study can provide insightful evidence to the field of sustainability development research. However, the data were collected in about 2016, and most of the data appear relatively old and might be subject to estimation bias caused by the technological gap in different time periods. Therefore, using more recent and comprehensive datasets in the future is recommended for investigating the association between economic growth, environmental conservation, and education.

## Conclusion

This study employed a large-scaled original internet and face-to-face individual survey to show the impact of educational attainment on sustainable development. The original survey is derived from 37 nations with 100,956 valid observations collected using individuals’ sustainable lifestyle as well as socio-economic and demographic background. We confirmed the relationship between educational attainment and household income by country and explored the association between education and sustainable lifestyle. The results are expected to provide insightful evidence to policymakers.

## Supplementary Information

Supplementary Information.

## Data Availability

The data is available upon reasonable request to the authors Xiangdan Piao or Shunsuke Managi.
