# Supplementary material for: The international role of education in sustainable lifestyles and economic development
Source: Sci Rep. 2023 May 30;13:8733. doi: 10.1038/s41598-023-35173-w (PMC10229545; doi:10.1038/s41598-023-35173-w)
Supplement: Supplementary file 1 — Supplementary Information. [file 41598_2023_35173_MOESM1_ESM.docx]

**Appendices**

**Table A1.** Descriptive statistics

| Variable | Mean | Std. Dev. |
| --- | --- | --- |
| *Education* |  |  |
| Junior school or lower | 0.08 | 0.27 |
| Senior high school | 0.18 | 0.39 |
| Vocational school | 0.10 | 0.30 |
| University | 0.51 | 0.50 |
| Graduate school | 0.11 | 0.31 |
| Household income | 19342.39 | 22510.90 |
| *Occupation* |  |  |
| Unemployed | 0.09 | 0.28 |
| Company owner | 0.02 | 0.14 |
| Government employee | 0.03 | 0.18 |
| Professional | 0.03 | 0.18 |
| Full-time employee | 0.45 | 0.50 |
|  |  |  |
| Part-time employee | 0.08 | 0.27 |
| Self-employed | 0.08 | 0.27 |
| Student | 0.06 | 0.23 |
| Homemaker | 0.08 | 0.28 |
| Other | 0.08 | 0.27 |
| *House status* |  |  |
| Rent | 0.26 | 0.44 |
| Owner | 0.71 | 0.45 |
| Other | 0.03 | 0.17 |
| One child | 0.29 | 0.46 |
| Two children | 0.24 | 0.43 |
| Three children or more | 0.12 | 0.33 |
|  |  |  |
| Female dummy | 0.49 | 0.50 |
| Number of family member | 3.26 | 1.58 |
| Age | 42.86 | 14.81 |
| Obs. | 92,128 | 92,128 |

**Table A2.** Survey information

| Country name | Start time | End time | Survey type | Number of deliveries | Observation |
| --- | --- | --- | --- | --- | --- |
| Japan | 2015/7/14 | 2015/8/5 | Internet | * | 11167 |
| Thailand | 2015/7/18 | 2015/7/23 | Internet | * | 1127 |
| Malaysia | 2015/7/23 | 2015/7/29 | Internet | * | 1106 |
| Indonesia | 2015/7/18 | 2015/7/23 | Internet | * | 2210 |
| Singapore | 2015/7/15 | 2015/7/21 | Internet | * | 587 |
| Vietnam | 2015/7/18 | 2015/7/28 | Internet | * | 1541 |
| Philippines | 2015/7/15 | 2015/7/22 | Internet | * | 1686 |
| Mexico | 2015/7/24 | 2015/7/27 | Internet | * | 1678 |
| Venezuela | 2015/7/24 | 2015/8/5 | Internet | * | 827 |
| Chile | 2015/7/24 | 2015/7/28 | Internet | * | 1192 |
| Brazil | 2015/7/23 | 2015/7/26 | Internet | * | 2298 |
| Colombia | 2015/7/24 | 2015/7/27 | Internet | * | 1115 |
| South Africa | 2015/7/15 | 2015/7/23 | Internet | * | 1123 |
| India | 2015/7/25 | 2015/8/11 | Internet | * | 5200 |
| Myanmar | 2015/7/6 | 2015/8/10 | Face to face | - | 1083 |
| Indonesia | 2015/6/30 | 2015/8/11 | Face to face | - | 202 |
| Vietnam | 2015/6/24 | 2015/6/30 | Face to face | - | 200 |
| India | 2015/7/21 | 2015/8/25 | Face to face | - | 1500 |
| Kazakhstan | 2015/8/25 | 2015/9/24 | Face to face | - | 1000 |
| Mongolia | 2015/8/19 | 2015/9/3 | Face to face | - | 500 |
| Egypt | 2015/9/14 | 2015/10/27 | Face to face | - | 1016 |
| Russia | 2015/8/31 | 2015/9/14 | Internet | * | 2221 |
| China | 2016/1/12 | 2016/2/29 | Internet | * | 20744 |
| Australia | 2016/2/10 | 2016/2/22 | Internet | * | 2029 |
| United States | 2016/8/16 | 2016/8/28 | Internet | 135,071 | 10683 |
| Germany | 2016/8/26 | 2016/9/7 | Internet | 19,727 | 3165 |
| United Kingdom | 2016/8/16 | 2016/8/28 | Internet | 34,978 | 2993 |
| France | 2016/8/26 | 2016/9/7 | Internet | 13,196 | 2138 |
| Spain | 2016/8/26 | 2016/9/7 | Internet | 17,665 | 2116 |
| Italy | 2016/8/29 | 2016/9/10 | Internet | 17,357 | 2106 |
| Sweden | 2016/8/31 | 2016/9/12 | Internet | 11,285 | 1330 |
| Canada | 2016/9/1 | 2016/9/13 | Internet | 9,478 | 1333 |
| Netherlands | 2016/8/29 | 2016/9/10 | Internet | 8,599 | 1371 |
| Greece | 2016/8/31 | 2016/9/12 | Internet | 6,601 | 1382 |
| Turkey | 2017/3/7 | 2017/3/20 | Internet | 33,719 | 2120 |
| Hungary | 2017/3/8 | 2017/3/15 | Internet | 29,355 | 1354 |
| Poland | 2017/3/8 | 2017/3/17 | Internet | 44,385 | 2227 |
| Czechia | 2017/3/8 | 2017/3/16 | Internet | 25,173 | 1400 |
| Romania | 2017/3/8 | 2017/3/18 | Internet | 29,219 | 1386 |
| Sri Lanka | 2017/3/9 | 2017/3/30 | Face to face | - |  |
